# Global trends of big data analytics in health research: a bibliometric study

**DOI:** 10.3389/fmed.2025.1456286

**Published:** 2025-07-01

**Authors:** Li Yao, Yan Liu, Tingrui Wang, Chunyan Han, Qiaoxing Li, Qinqin Li, Xiaoli You, Tingting Ren, Yinhua Wang

**Affiliations:** ^1^Department of Respiratory and Critical Care Medicine, The Affiliated Hospital of Guizhou Medical University, Guiyang, China; ^2^School of Management & Collaborative Innovation Laboratory of Digital Transformation and Governance, Guizhou University, Guiyang, China; ^3^School of Nursing, Guizhou Medical University, Guiyang, China; ^4^Emergency Critical Care Unit, Qingdao Municipal Hospital Group, Qingdao, China; ^5^Department of Hepatobiliary Surgery, The Affiliated Hospital of Guizhou Medical University, Guiyang, China; ^6^Department of Nursing, The Affiliated Hospital of Guizhou Medical University, Guiyang, China

**Keywords:** big data, health, bibliometric study, VOSviewer, CiteSpace

## Abstract

**Background:**

The field of “Big Health,” which encompasses the integration of big data in healthcare, has seen rapid development in recent years. As big data technologies continue to transform healthcare, understanding emerging trends and key advancements within the field is essential.

**Methods:**

We retrieved and filtered articles and reviews related to big data analytics in health research from the Web of Science Core Collection, including SCI Expanded and SSCI, covering the period from 2009 to 2024. Bibliometric and co-citation analyses were conducted using VOSviewer and CiteSpace.

**Results:**

A total of 13,609 papers were analyzed, including 10,702 original research and 2,907 reviews. Co-occurrence word analysis identified six key research areas: (1) the application of big data analytics in health decision-making; (2) challenges in the technological management of health and medical big data; (3) integration of machine learning with health monitoring; (4) privacy and ethical issues in health and medical big data; (5) data integration in precision medicine; and (6) the use of big data in disease management and risk assessment. The co-word burst analysis results indicate that topics such as personalized medicine, decision support, and data protection experienced significant growth between 2015 and 2020. With the advancement of big data technologies, research hotspots have gradually expanded from basic data analysis to more complex application areas, such as the digital transformation of healthcare, digital health strategies, and smart health cities.

**Conclusion:**

This study highlights the growing impact of big data analytics in healthcare, emphasizing its role in decision-making, disease management, and precision medicine. As digital transformation in healthcare advances, addressing challenges in data integration, privacy, and machine learning integration will be crucial for maximizing the potential of big data technologies in improving health outcomes.

## 1 Introduction

Big data involves the comprehensive analysis and processing of all available data, avoiding the simplifications inherent in random sampling surveys. Big data is traditionally defined by five characteristics: volume, velocity, variety, value, and veracity (1). Advancements in technology and deeper applications have broadened the characteristics of big data to include variability, visualization, verifiability, value density, and viability (2). These characteristics not only highlight the challenges associated with volume and velocity in big data but also emphasize the potential to extract accurate, reliable, and valuable information from complex and variable data sets (3). As information technology continues to advance rapidly, big data has permeated various aspects of life, establishing an increasingly close connection with health. The emergence of the big data era presents an opportunity to manage dynamic health conditions, address health issues promptly, and develop personalized medical strategies (4). Clearly, health and medical big data have become critical areas of research.

Health and medical big data encompass all data related to medical care and health outcomes generated throughout the medical process (5). “This includes electronic health records, medical monitoring records, biometric data, public health information, and health insurance data. In healthcare and medical fields, big data can be extensively utilized for clinical decision support, pharmaceutical development, disease monitoring, and health management. This utilization involves various big data analytics techniques, including data structuring, image analysis, and intelligent detection (6). Consequently, the technologies associated with the application of big data are crucial to advancing the healthcare industry.

Recent studies have extensively explored the transformative role of big data analytics in medical practice. For instance, Lorenzo et al. (7) emphasized the transformative potential of big data in predicting oncology patient outcomes and enhancing personalized treatment. Another pivotal study by Dong et al. (8) demonstrated how big data facilitates real-time epidemic tracking, significantly aiding rapid and effective public health responses. Furthermore, Kindle et al. (9) explored the incorporation of big data analytics into clinical decision-making systems, discovering that data-based models significantly enhanced diagnostic precision and the efficiency of patient care. These studies highlight the essential role of advanced analytics in improving medical services and outcomes. Despite advancements, further detailed analyses and updates on the global implementation of big data and its long-term impact across various medical fields are still needed. Our research aims to address this issue through bibliometric methods.

Bibliometric research, which analyzes the characteristics of literature, serves as a technique for examining the distribution structure, quantitative relationships, and evolutionary patterns of relevant information within publications. This approach is used to assess research output and trends across various fields (10). VOSviewer, a Java-based software, enables the construction and visualization of bibliometric networks, such as citation coupling, co-citation analysis, author co-citation, and co-occurrence word analysis based on scientific publications (11). Bibliometric analysis using VOSviewer has been applied in various medical fields, including surgery (12), oncology (13), and nutrition (14), to gain deeper insights. Several bibliometric studies focusing on big data have been published, addressing topics such as infectious diseases (15), HIV (16), and critical care (17). Two studies have focused on bibliometric research in the healthcare industry using big data, analyzing articles published before 2016 (6, 18). Big data research in healthcare has garnered significant attention both domestically and internationally, prompting more researchers to use big data analytics tools to address medical issues. Since 2016, there has been a growing number of studies on the application of big data technologies in healthcare, which require further exploration through bibliometric analysis (19). In addition, CiteSpace is used to detect the knowledge structure, the evolution of research hotspots, and the burst trends of citations in the literature. This study aims to employ VOSviewer and CiteSpace for an updated and comprehensive analysis of publications on health big data analytics.

## 2 Materials and methods

### 2.1 Study design

A bibliometric analysis was conducted using VOSViewer version 1.6.19 and CiteSpace 6.2.R4 to investigate research on big data analytics in healthcare. The bibliometric methodology involved d five stages: study design, data collection, data analysis, data visualization, and interpretation (20).

### 2.2 Search strategy

Two investigators independently conducted a literature search. We searched the Web of Science (WoS) Core Collection, including SCI Expanded and SSCI, for studies published between 2009 and November 28, 2024. WoS was selected because of its extensive use in bibliometric studies and its superior coverage of high-impact journals (21). The search strategy employed was “TS = Topic.” The search formulas used were TS = (big data) and TS = (health OR healthcare OR clinical OR medical OR medicine OR medical care).

### 2.3 Screening strategy

**Figure 1:**
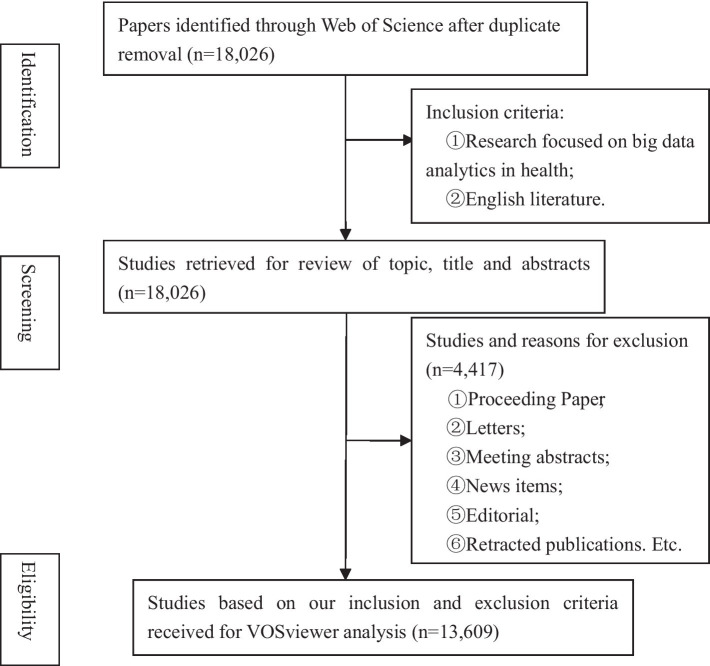
Flowchart of the preferred reporting items for VOSviewer analysis.

We excluded non-English articles, duplicate literature, letters, meeting abstracts, news items, editorials, comments, and retracted publications. There were no restrictions on publication date. Figure 1 illustrates the process and results of literature screening. A total of 13,609 documents were included in the analytic sample.

### 2.4 Data extraction

The extracted literature information for visualization and bibliometric analysis includes the publication year, journal title, authorship, WoS category, manuscript type, publication country/region, publication organization, total citations, and H5-index.

### 2.5 Statistical analysis

The exported bibliographic file was first imported into Occurrence 14.9 software for deduplication, data cleaning, and synonym consolidation. Subsequently, information such as publication date, authors, institutions, journals, and keywords was extracted. Bibliometric methods utilize mathematics, statistics, and philology to quantitatively analyze elements such as journal titles, publication years, countries/regions, organizations, authorship, citation counts, and H5-index. This study employed VOSviewer (version 1.6.19) to map keyword co-occurrence, citations, publications, bibliographic coupling in countries and institutions, as well as thematic and trend topic networks. CiteSpace 6.2.R4 was also used to detect bursts in keywords and references.

## 3 Results

### 3.1 Number of publications and trend analysis

**Figure 2:**
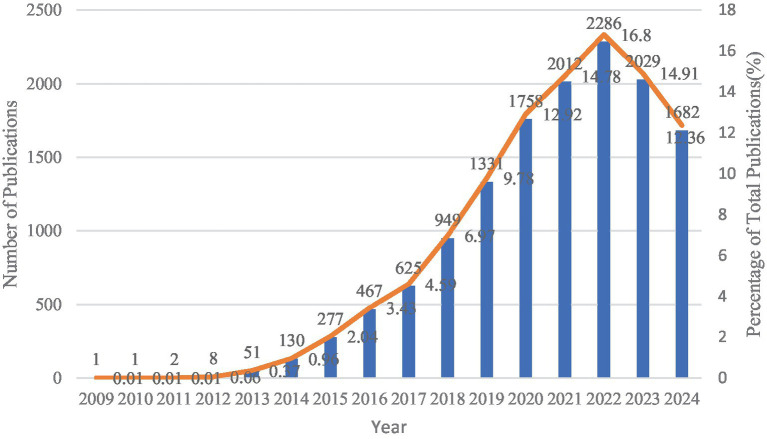
The annual publications on big data analytics in health from 2009 to 2024.

Among the 13,609 articles included, 10,702 (78.6%) were original articles, and 2,907 (21.4%) were reviews. Scholars have published articles on the application of big data technology in the medical industry since 2009. Since 2014, literature on big data applications in this field has shown annual growth. The number of papers published in 2022 reached its peak at 2,286, which is 36.3 times greater than that published in 2013 and earlier. Polynomial fitting yielded an *R^2^* value of 0.893, indicating a significant correlation between the year of publication and the annual number of publications. This trend suggests that literature in this field is likely continue to expand. The application of big data analytics in the medical field remains a focal point of research. Figure 2 presents the contents.

### 3.2 Analysis of countries/regions

**Figure 3:**
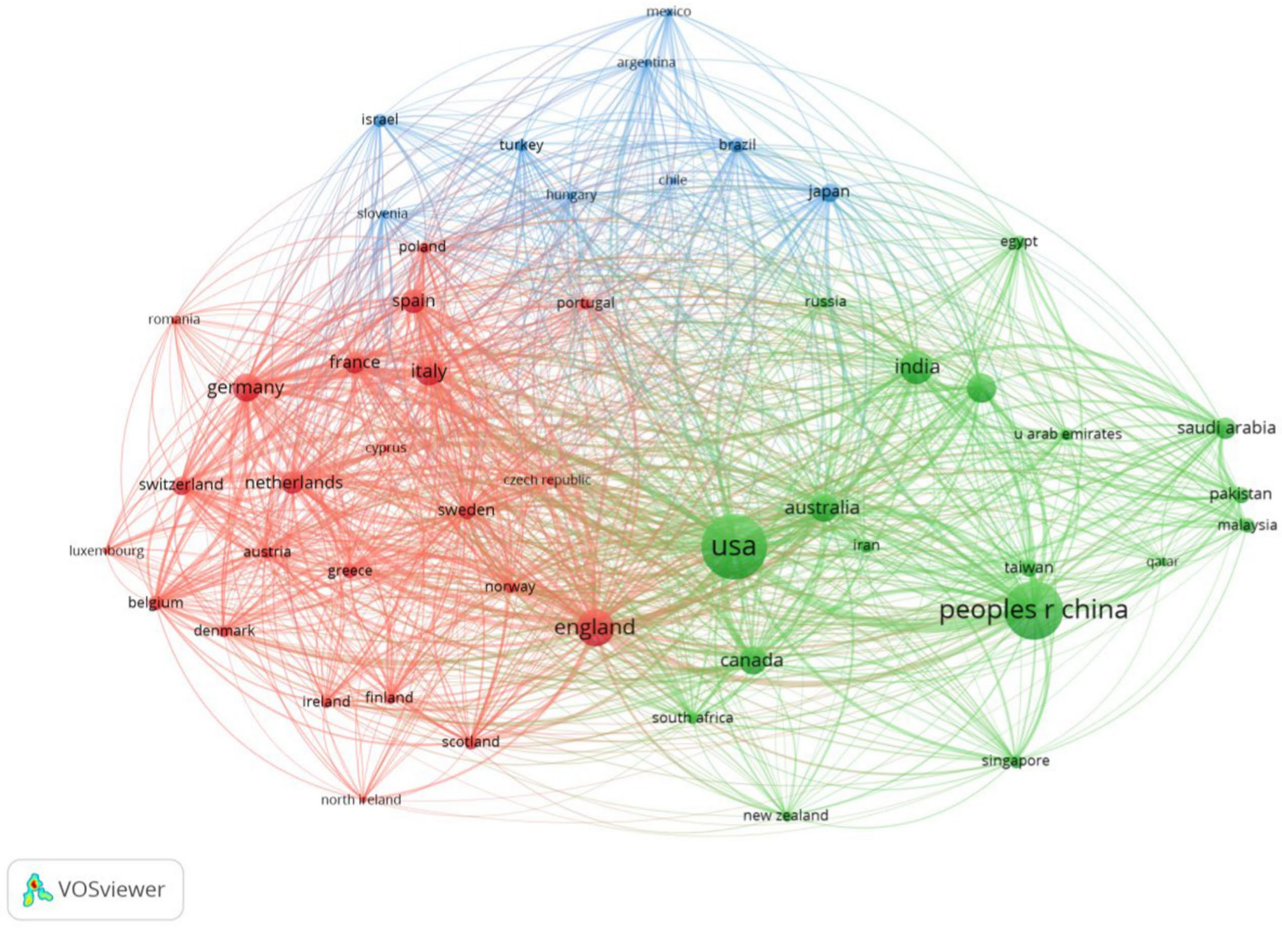
A visualization network of collaboration between authors’ countries/regions on big data analytics in health.

The literature in this field originates from 149 countries/regions. The USA and China have each published more than 3,000 articles, and 97 countries/regions have published more than five articles. Table 1 summarizes the top 10 countries/regions. The most prolific country was the USA, with 4,053 publications on medical big data analytics, which also had the highest total citations (159,464). Following China with 3,184 publications, England ranked third with 1,341 publications and the highest average citation rate of 40.06. The collaboration between countries and regions was analyzed using VOSviewer software (Figure 3). The lines between nodes indicate a cooperative relationship; the thicker the lines, the closer the relationship, denoting a stronger total link strength (TLS). The countries with the highest TLS were the USA (3,957), England (3,059), China (2,065), Germany (1,852), and Italy (1,705).

**Table 1:** The main countries/regions, and institutions contributing to publications on big data analytics in health.

Rank	Country/Region	Counts	Total citation	Average citation	Total link strength
1	USA	4,053	159,464	39.34	3,957
2	China	3,184	82,042	25.77	2,065
3	England	1,341	53,716	40.06	3,059
4	India	1,095	26,317	24.03	1,350
5	South Korea	827	19,231	23.25	784
6	Germany	770	21,436	27.84	1,852
7	Italy	759	20,870	27.50	1,705
8	Australia	753	27,097	35.99	1,637
9	Canada	735	27,569	37.51	1,374
10	Spain	535	13,270	24.80	1,288

### 3.3 Analysis of institutions

**Figure 4:**
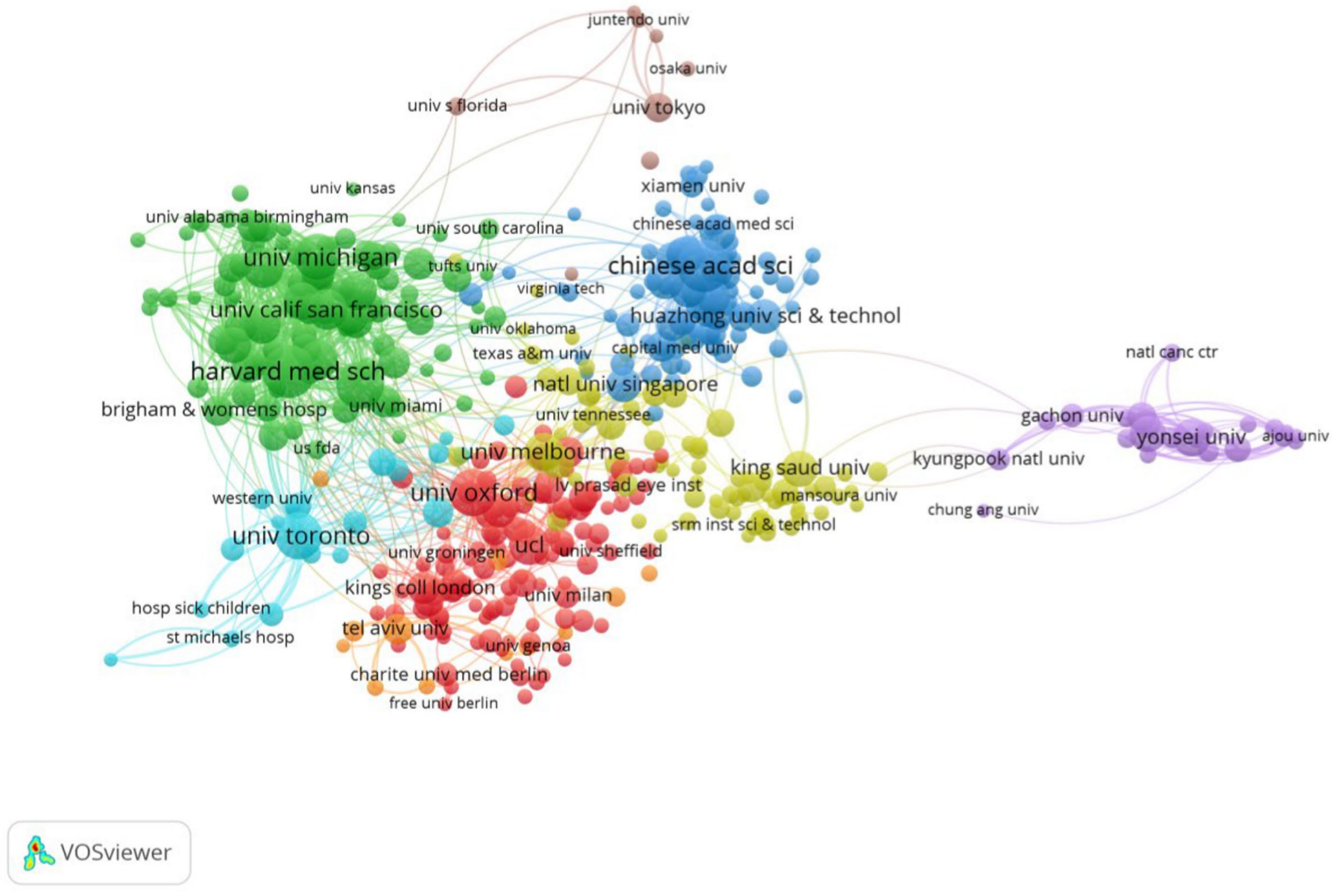
A visualization network of collaboration between authors’ institutions on big data analytics in health.

Of the 13,959 institutions involved in research on big data analytics in medicine, 1,436 had published more than 5 papers. Table 2 presents the publications of the top 10 institutions over the last 15 years. Harvard Medical School (211 articles), the Chinese Academy of Sciences (209 articles), and Stanford University (169 articles) were the top three institutions in terms of published articles. The interinstitutional cooperation network map for institutions with 15 or more publications was generated using VOSviewer software (Figure 4). The leading institutions by TLS were Harvard Medical School (755), the University of Toronto (602), and the University of California, San Francisco (499).

**Table 2:** The main institutions contributing to publications on big data analytic in health.

Rank	Institutions	Country/Region	Counts	Total citation	Average citation	Total link strength
1	Harvard Medical School	USA	211	9,011	42.71	755
2	Chinese Academy of Sciences	China	209	9,921	47.47	428
3	Stanford University	USA	169	7,636	45.18	477
4	University of Oxford	England	168	9,545	56.82	460
5	University of Toronto	Canada	167	7,572	45.34	602
6	University of Michigan	USA	160	5,629	35.18	465
7	University of California, San Francisco	USA	124	5,811	46.86	499
8	University of Pennsylvania	USA	123	5,504	44.75	487
9	University of California, Los Angeles	USA	121	3,493	28.87	354
10	University of Melbourne	Australia	119	4,456	37.45	366

### 3.4 Journal analysis

A total of 13,609 articles were published in 3,973 journals, with 573 journals publishing more than 5 articles each. The top 10 journals published 1,397 articles, representing 10.3% of the total. IEEE Access led in both publication count (321 articles) and citations (14,842), significantly outperforming other journals. Future Generation Computer Systems-The International Journal of eScience, which had the highest impact factor in 2023 (6.2), ranked tenth among the top 10 journals with the most publications (Supplementary Table S1). A network visualization map, illustrating the bibliometric coupling of journals with a minimum contribution of five articles, is presented in Supplementary Figure S1. PLoS One (8,186 co-citations), IEEE Access (7,621 co-citations), and Nature (6,721 co-citations) were the three most frequently co-cited journals (Supplementary Table S2). The map illustrates the distribution of 158 highly co-cited journals, each with a minimum of 600 co-citations, serving as a hotspot map for co-cited journals (Supplementary Figure S2).

### 3.5 Analysis of authors

The field included 55,858 authors and 337,287 co-cited authors. Table 3 presents the top 10 most productive, highly cited, and co-cited authors. Anthony Vipin Das authored the most articles (22), followed by Wei Wang (23), and finally M. Shamin Hossain and Kyungyong Chung, each with 22. Twenty-four research clusters were identified when mapping research networks for 595 authors who had produced at least five documents (Supplementary Figure S3). Khoshgoftaar, Taghi M. received the highest number of citations (6,247), significantly more than others. The World Health Organization (1,192) was the most frequently co-cited author, followed by Chen, M. (792) and Zhang, Y. (653). Author co-citation maps were created using 1,695 authors, each cited more than 35 times (Supplementary Figure S4).

**Table 3:** Top 10 productive authors and co-cited authors in the field of big data analytics in health.

Rank	Author	Counts	Cited author	Citations	Co-cited author	Citations
1	Das, Anthony Vipin	37	Khoshgoftaar, Taghi M.	6,247	World Health Organization	1,192
2	Wang, Wei	23	Chen, Min	2,018	Chen, M.	792
3	Chung, Kyungyong	22	Wang, Peng	1,679	Zhang, Y.	653
4	Hossain, M. Shamim	22	Guizani, Mohsen	1,626	Liu, Y.	623
5	Bragazzi, Nicola Luigi	21	Hossain, M. Shamin	1,433	Wang, Y	550
6	Wang, Lei	21	Zhang, Zhongheng	1,392	Obermeyer, Z.	491
7	Chen, Bin	20	Gadekallu, Thippa Reddy	1,384	Raghupathi, W.	473
8	Rodrigues, Joel J. P. C.	19	Yang, Laurence T.	1,364	Lee, J.	464
9	Wang, Hao	18	Muhammad, Ghulam	1,311	Li, Y.	441
10	Kumar, Neeraj; Li, Li; Zhang, Lei	16	Chen, Bin	1,310	Kim, J.	428

### 3.6 Analysis of cited references

A total of 613,873 references were found, of which 1,153 were cited more than 20 times. We displayed the top 10 articles with the highest number of citations. The top co-cited reference is by Raghupathi W. with 440 citations, followed by Murdoch T. B. with 342 citations, and Obermeyer Z. with 323 citations (Supplementary Table S3). As shown in Supplementary Figure S5, the 25 references with the greatest number of citation bursts date back to 2014. Significantly, seven citations remain in burst mode even today.

### 3.7 Co-occurrence analysis and clustering analysis of keywords

**Figure 5:**
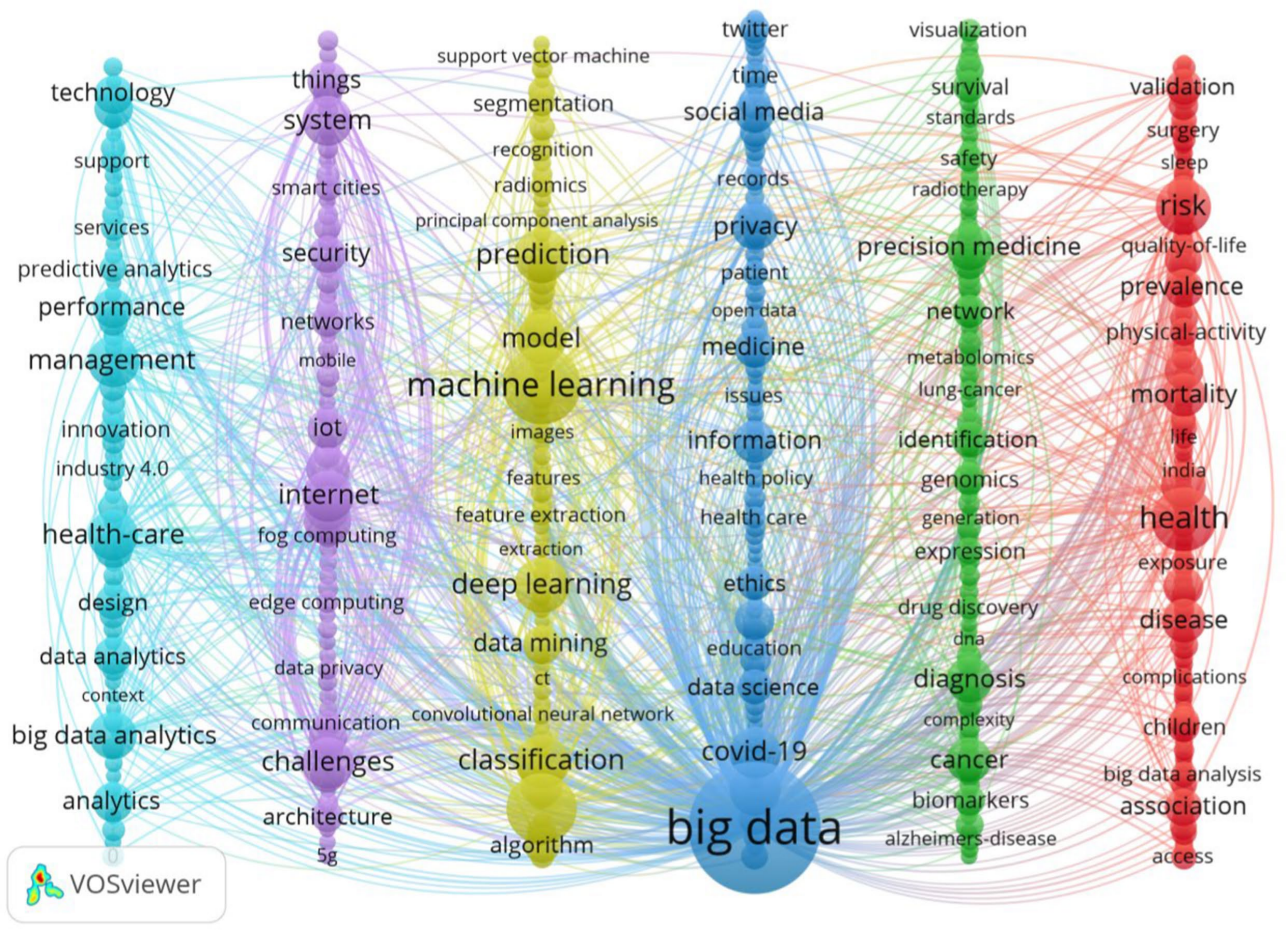
Co-occurrence mapping of keywords.

A total of 3,536 keywords were identified, with a cumulative frequency of 40,467 instances. Table 4 displays the 20 most frequently occurring keywords, each with an occurrence frequency of more than 450. IA keyword co-occurrence diagram was created in this domain using VOSviewer, highlighting 552 keywords that appeared more than 30 times for visualization (Figure 5). Each color in the diagram represents a cluster indicating a similar subject among the publications, and each keyword is represented by a circle. The keywords were categorized into five clusters: Cluster 1 (light blue) encompassed healthcare, management, big data analytics, and performance; Cluster 2 (purple) centered on systems, the internet, cloud computing, healthcare, and challenges; Cluster 3 (yellow) highlighted machine learning, artificial intelligence, deep learning, prediction, and classification; Cluster 4 (dark blue) encompassed big data, information, privacy, and ethics; Cluster 5 (green) covered precision medicine, identification, and diagnosis; Cluster 6 (red) centered on big data analytics, risk, and mortality. The thematic terms for keyword clustering are presented in Table 5. VOSviewer color-coded the keywords on the map according to their average year of appearance (Supplementary Figure S6). The prominence of artificial intelligence and deep learning in medical big data analytics is underscored by their frequent mention in recent discussions.

**Table 4:** High-frequency keywords in the studies of big data analytics in health.

Rank	Keywords	Frequency	Total link strength
1	big data	6,170	28,869
2	machine learning	1,671	9,408
3	artificial intelligence	1,287	7,487
4	health	1,046	5,148
5	risk	700	3,702
6	deep learning	693	3,811
7	prediction	693	4,296
8	classification	678	3,910
9	COVID-19	607	2,810
10	challenges	606	4,029
11	internet	601	4,248
12	management	590	3,582
13	care	582	3,364
14	system	567	3,247
15	model	565	3,130
16	health-care	546	3,214
17	framework	493	3,331
18	big data analytics	479	2,584
19	diagnosis	458	2,635
20	precision medicine	454	2,468

**Table 5:** Detailed list of clustered keywords and thematic terms.

Cluster	Theme	Representative keywords
#1	Application of big data analytics in health decision-making	Health care; management; decision making; technology; support; supply chain; services; innovation; predictive analytics; performance
#2	Challenges in the technological management of health and medical big data	Internet; IoT; fog computing; edge computing; 5G; mobile; systems; challenges; data privacy; big data applications; data management; medical big data
#3	Integration of machine learning with health monitoring	Machine learning; deep learning; data mining; algorithm; feature extraction; model; prediction; classification; prognostics; health monitoring
#4	Privacy and ethical issues in health and medical big data	Big data; data science; social media; health information; electronic health record; digital health; patient; medicine; ethics; health policy; data protection
#5	Data integration and application in precision medicine	Data integration; precision medicine; treatment; diagnosis; prognosis; survival; cancer; Alzheimer’s disease; genomics; DNA; biomarkers
#6	Application of big data in disease management and risk assessment	Big data analysis; health; disease; administrative data; prevention; risk; prevalence; exposure; complications

### 3.8 Burst of keywords

**Figure 6:**
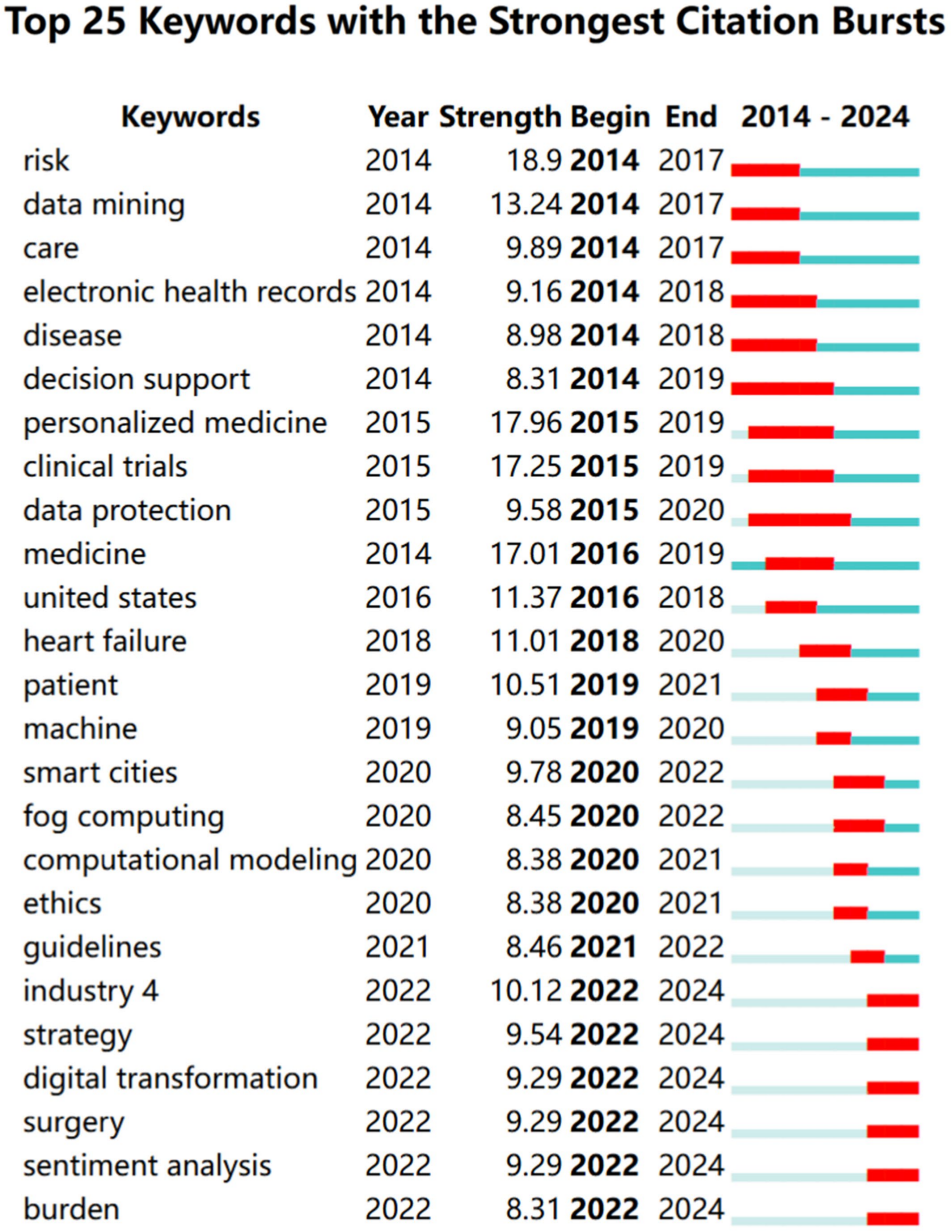
Top 25 keywords with the strongest citation bursts.

Burst keywords are terms that frequently emerge over a period of time. Figure 6 highlights the top 25 keywords with the most significant citation bursts. The keyword “personalized medicine” had the highest burst value (*n* = 17.96), while “decision support” and “data protection” exhibited the longest burst periods. The keywords that experienced the most significant bursts from 2022 to 2024 included Industry 4.0, strategy, digital transformation, surgery, sentiment analysis, and burden.

## 4 Discussion

This study employed two prominent bibliometric analysis tools, VOSviewer and CiteSpace, to examine the evolution of big data research in the health sector over the past 15 years. Our analysis provided a thorough overview of the current landscape, developmental trends, and emerging research hotspots, providing valuable insights for researchers to enhance their understanding of the dynamics and future prospects of this field. The findings reveal that 71.8% of the published literature in the past 5 years underscores the rapid growth of big data applications in the medical domain. The reasons for this rapid growth may include the introduction of policies by various countries and regions to promote digital health and precision medicine, the rapid development of big data analytics technologies, and the continuous deepening of interdisciplinary collaboration. Furthermore, the outbreak of COVID-19 in 2020 prompted a substantial volume of literature focusing on how to leverage big data technologies to address pandemic challenges, such as real-time epidemic data analysis, vaccine distribution, and public health decision-making support. However, it should be noted that the expansion of the Web of Science Core Collection may have a potential impact on the growth of publication numbers (24).

The USA occupies a leading position in global health big data research, as evidenced by 4,053 published papers and a total citation count of 159,464, underscoring the country’s prominent role in advancing this field. Additionally, the average citation count of USA research ranks third globally, indicating that the country excels not only in volume but also in academic impact. This trend is further supported by the USA’s TLS of 3,957, highlighting its significant role in international collaborations. In comparison, China and England closely follow, with 3,184 and 1,341 publications, respectively. Notably, the average citation rate of 40.06 for England reflects the high global reputation of its research.

Through the analysis of international collaboration, we found that the USA, England, and China are core participants in the global network, a fact further supported by their strong TLS values. The collaboration network diagram generated by VOSviewer shows that these three countries occupy central positions with close cooperation ties. The increasing collaborative efforts among nations further highlight the globalization of health big data research, indicating substantial cross-border cooperation in addressing common healthcare challenges. At the institutional level, Harvard Medical School, a prestigious medical school within Harvard University, stands as a key hub in this field, having published 211 papers and leading in centrality indicators such as link strength and intermediary centrality. Six top institutions from the USA rank among the top 10, further emphasizing the country’s dominant position in health big data research. The Chinese Academy of Sciences and Stanford University follow closely in terms of publication volume, showcasing the global influence of these institutions. The TLS values of leading institutions, such as Harvard Medical School (755), the University of Toronto (602), and the University of California, San Francisco (499), demonstrate their active engagement in interdisciplinary collaborations, particularly in research areas requiring large-scale data analysis. This underscores the growing significance of big data in the healthcare sector.

We observed that the top three journals have published more than 150 articles each, with impact factors exceeding 3, and are classified as Q1 or Q2 in the Journal Citation Reports (JCR) rankings. This not only indicates the high quality of research and academic influence of these journals in the field of health big data, but also reflects researchers’ recognition of these journals as core platforms for knowledge dissemination. Consequently, future research should consider submitting to these high-impact journals to enhance the visibility and citation potential of their studies.

The 10 most-cited articles illuminate the primary research directions in health big data analytics, emphasizing the introduction of methodologies and risk identification. For instance, Raghupathi W. systematically discusses the structure, implementation strategies, and challenges of healthcare big data in his highly co-cited paper, “Big data analytics in healthcare: promise and potential” (23), which lays the groundwork for subsequent research in the field. Similarly, Murdoch T. B.’s review in The Journal of the American Medical Association highlights the significance and inevitability of big data technologies in healthcare (2013) (25). These highly cited papers are predominantly review articles that examine the methodological frameworks of big data in healthcare, while high-quality original research on practical applications remains relatively scarce. Co-citation analysis reveals the dynamic evolution of research frontiers. Based on the temporal analysis of the 25 most co-cited papers, early research focused on fundamental methods for big data in medicine, including data collection, storage, and processing techniques. However, recent research trends have gradually shifted toward application areas such as disease risk identification and precision medicine (26, 27). Notably, the latest research has concentrated on the integration of big data technologies with artificial intelligence and machine learning algorithms, as well as improvements in data privacy protection measures (28, 29). This trend reflects the significant attention from both academia and industry toward the potential of big data technologies in healthcare. Furthermore, an author analysis reveals that prolific authors such as Anthony Vipin Das and Wei Wang have played crucial roles in advancing the field of health big data, while Taghi M. Khoshgoftaar has demonstrated substantial academic influence through his high citation count. Additionally, the World Health Organization, as a highly co-cited author, further highlights its central role in policy development and the application of health data.

The results from topic clustering indicate that research hotspots in healthcare big data analytics are characterized by significant diversity and interdisciplinary features. For instance, Cluster 1 emphasizes healthcare management and decision support, demonstrating the potential of big data to optimize resource allocation and improve service efficiency. In contrast, Cluster 3 centers on artificial intelligence technologies, such as machine learning and deep learning, underscoring their critical roles in health monitoring, disease prediction, and classification. With ongoing advancements in medical devices, internet connectivity, and cloud computing, vast amounts of heterogeneous big data are generated daily within the healthcare sector (30). Transforming these vast data resources into actionable knowledge bases to improve the efficiency and quality of healthcare services has become a crucial task for professionals in the field. By employing advanced data modeling techniques, such as artificial intelligence, machine learning, and deep learning, it is possible to effectively identify risk factors for patients, thereby enabling the prediction and management of high-risk populations (4). With the increasing availability of genomic data, these technologies offer a solid scientific foundation for early disease prediction, precision treatment, and personalized healthcare. For example, big data technologies have shown significant potential and value in the diagnosis and risk prediction of cardiovascular diseases (31), respiratory diseases (32), sepsis (33), and cancer (34). By enhancing the analytical capabilities of unstructured data and leveraging advanced big data technologies, clinical decision support systems can thoroughly analyze medical imaging data and extract key information from medical literature, thus constructing comprehensive medical expert knowledge bases. This technological support not only aids physicians in making more accurate diagnoses but also provides recommendations for drug dosage adjustments and personalized treatment plans.

The keywords “privacy” and “ethics” in Cluster 4 underscore the growing concerns regarding data security and privacy protection as healthcare data application expands. This suggests that the use of big data in the healthcare sector encounters numerous challenges (35). The primary topic clustering in Cluster 2, which pertains to the challenges of implementing big data technologies such as the Internet of Things (IoT) in healthcare, further reinforces this assertion. A major challenge lies in ensuring the security of healthcare information, particularly when it involves sensitive patient data and personal privacy (36). Moreover, despite the abundance of available healthcare data, effectively integrating and improving the overall quality of this data remains a considerable challenge (37). Currently, there is a shortage of professionals who possess both medical expertise and advanced big data analysis skills, including machine learning, artificial intelligence, and deep learning. This deficiency is insufficient to meet the health needs of the broader population. Consequently, this relative shortage of talent restricts the efficiency of data management and somewhat hinders the effectiveness of data sharing (22). Therefore, it is imperative to continue accelerating the training of interdisciplinary experts who possess both medical backgrounds and advanced big data analysis skills (38).

The keyword color distribution over time indicates a gradual shift in recent research trends towards areas like artificial intelligence and precision medicine. For instance, the higher average values of keywords like “artificial intelligence” and “deep learning” suggest that these technologies have emerged as key research hotspots in healthcare big data in recent years. Concurrently, the keywords “precision medicine” and “diagnosis” in Cluster 5 reflect the healthcare sector’s accelerating transition from traditional broad-spectrum treatments to personalized and precision-based approaches, supported by big data-related technologies. Notably, during the global COVID-19 pandemic, big data management and analysis technologies played a critical role, demonstrating their vast potential in infectious disease monitoring and control (39). These technologies are essential for the rapid detection and prevention of infectious disease spread, further underscoring the importance of big data in public health emergency management. Additionally, the persistent emergence of keywords like “decision support” and “data protection” highlights the ongoing importance of these themes in healthcare big data analytics, aligning with the increasing trend of utilizing healthcare big data in real-time decision support systems. From 2022 to 2024, the notable rise of keywords like “Industry 4.0,” “digital transformation,” and “sentiment analysis” suggests that the healthcare sector is accelerating its integration with other cutting-edge technologies, including IoT and natural language processing, which are crucial for advancing the development of the broader health industry. Furthermore, the development of “smart healthcare” or “digital health” focuses on leveraging technology to enhance medical services, improve patient care, and enable real-time health monitoring. The theoretical frameworks of “data integration,” “data collaboration,” and others have become key components in the application of big data in health management, ensuring that data from various health domains can seamlessly work together (40). Overall, the rise of hot topics like artificial intelligence, precision medicine, digital transformation and privacy ethics reflects both the technology-driven changes at the academic frontier and the challenges and needs faced by the healthcare sector in practical applications. However, there are certain limitations in the current research, including the insufficient exploration of regional differences in research topics. Future studies could further investigate the diversification and localization characteristics of global healthcare big data applications. Moreover, how to balance the ethical risks of big data technologies with their societal benefits is an important direction that requires in-depth consideration.

This study updates the analysis of the most recently published articles to identify the current research trends in the application of big data in the healthcare sector. Before conducting the literature search, we reviewed several high-quality research papers, carefully extracted relevant keywords, and extensively consulted with domain experts to develop a comprehensive and scientifically sound search strategy. As a result, our search method is both rigorous and systematic, ensuring the accuracy and reliability of the findings. However, there are some inherent limitations in this bibliometric study. First, our analysis was limited to English-language articles from the WoS Core Collection database, which may have resulted in the exclusion of significant literature published in other languages or in journals not indexed by WoS Core Collection, potentially affecting the comprehensiveness of the study. This limitation is common in bibliometric research, primarily due to the incompatibility between databases when conducting comparative analyses, particularly regarding bibliometric impact metrics. These differences are largely attributable to the varying coverage of journals, conference proceedings, and books across different databases. Therefore, to ensure analytical consistency and facilitate a broader exploration of data fields and metadata, we confined our analysis to a specific database, the WoS Core Collection. Second, recent publications, particularly those from 2024, typically have fewer citations, as they may not yet have gained sufficient recognition yet. This could delay the identification of the latest research advancements. Moreover, citation counts can be influenced by various factors, including journal impact factors, author self-citations, incomplete references, and citation biases. As a result, some groundbreaking studies may initially receive limited citations until their significance is more broadly acknowledged. This phenomenon, known in bibliometrics as “forgotten through absorption,” has been widely discussed and recognized in previous research. Meanwhile, it is important to note that the WoS Core Collection has certain limitations when conducting historical literature retrieval. According to Liu (41), the WoS Core Collection faces challenges in retrieving older literature, including issues such as limitations in topic searches. As a result, the relatively low number of early publications in this study may be closely associated with these technical constraints. Finally, the inability to conduct a comprehensive funding analysis constitutes another limitation of this study. Future research could investigate this aspect further, thereby offering additional insights into the influence of financial support on the direction of research. Moreover, although bibliometric analysis is highly useful for identifying academic trends, it may not fully capture the long-term impact of big data in the healthcare sector. To address this issue, supplementary methods such as case studies, policy evaluations, and technology assessments could be considered to gain a deeper understanding of the enduring effects of big data in this field. Future research could further assess the sustained impact of big data technologies in healthcare through longitudinal studies.

## 5 Conclusion

We analyzed the application of big data in the health sector over the past 15 years using bibliometric tools including VOSviewer and CiteSpace. Our research indicates that big data technologies have substantial potential to enhance the accuracy of disease diagnostics and treatment outcomes, particularly in the realms of cancer treatment and disease risk assessment. However, we also identified several limitations within big data research, including constraints related to data sources and challenges in data processing. With advancements in data analysis technologies and the refinement of healthcare policies, big data is expected to play a more significant role in public health management, disease prevention, and health promotion. We recommend prioritizing interdisciplinary collaboration to integrate and apply big data technologies in healthcare, optimizing medical services, and improving public health outcomes.
