# Supplementary material for: Global trends of big data analytics in health research: a bibliometric study
Source: Front Med (Lausanne). 2025 Jul 1;12:1456286. doi: 10.3389/fmed.2025.1456286 (PMC12259701; doi:10.3389/fmed.2025.1456286)

**Supplementary Table S1** Publications and evaluation metrics of top 10 journals in term of publication volume.

| **Rank** | **Journal** | **Country/region** | **Number of**  **publication** | **Impact factor**  **(2023)** | **H5-index** | **JCR partition (2023)** | **Citation** |
| --- | --- | --- | --- | --- | --- | --- | --- |
| **1** | IEEE Access | USA | 321 | 3.4 | 56 | Q1 | 14,842 |
| **2** | Journal of Medical Internet Research | Canada | 159 | 5.8 | 116 | Q1 | 4,091 |
| **3** | International Journal of Environmental Research and Public Health | Switzerland | 155 | 4.6* | 78 | Q2 | 3,246 |
| **4** | PlOS One | USA | 137 | 2.9 | 268 | Q1 | 2,237 |
| **5** | Sensors | Switzerland | 123 | 3.4 | 132 | Q1 | 3,921 |
| **6** | Sustainability | Switzerland | 123 | 3.3 | 53 | Q2 | 2,075 |
| **7** | Frontiers in public health | Switzerland | 109 | 3.0 | 80 | Q2 | 929 |
| **8** | Applied Sciences-basel | Switzerland | 100 | 2.5 | 23 | Q3 | 1,209 |
| **9** | Scientific Reports | England | 97 | 3.8 | 149 | Q1 | 1,347 |
| **10** | Future Generation Computer Systems-the International Journal of eScience | Netherlands | 73 | 6.2 | 93 | Q1 | 4,789 |

* Impact factor 2021

**Supplementary Table S2** Co-citations and evaluation metrics of top 10 journals in term of co-citations.

| **Rank** | **Journal** | **Country/region** | **Co-citations** | **Impact Factor**  **(202**3**)** | **H**5**-index** | **JCR Partition (202**3**)** |
| --- | --- | --- | --- | --- | --- | --- |
| **1** | PlOS One | USA | 8,186 | 2.9 | 268 | Q1 |
| **2** | IEEE Access | England | 7,621 | 3.4 | 56 | Q1 |
| **3** | Nature | USA | 6,721 | 50.5 | 1,096 | Q1 |
| **4** | New England Journal of Medicine | USA | 6,020 | 96.2 | 933 | Q1 |
| **5** | Journal of the American Medical Association | USA | 4,815 | 63.1 | 622 | Q1 |
| **6** | Science | USA | 4,809 | 44.7 | 1,058 | Q1 |
| **7** | Scientific Reports | England | 4,764 | 3.8 | 149 | Q1 |
| **8** | Lancet | England | 4,167 | 89.4 | 700 | Q1 |
| **9** | [SENSORS](https://www.letpub.com.cn/index.php?page=journalapp&view=detail&journalid=7473) | SWITZERLAND | 3,780 | 3.4 | 132 | Q1 |
| **10** | Journal of the American Medical Informatics Association | England | 3,695 | 4.7 | 132 | Q1 |

**Supplementary Table S3** Detailed information on the top 10 references in term of co-citations.

| **Rank** | **References** | **Journal** | **First Author** | **Year** | **Co-citation** |
| --- | --- | --- | --- | --- | --- |
| **1** | Big data analytics in healthcare: promise and potential | Health Information Science and Systems | Raghupathi W | 2014 | 440 |
| **2** | The inevitable application of big data  to health care | JAMA - Journal of the American Medical Association | Murdoch TB | 2013 | 342 |
| **3** | Predicting the future-big data, machine learning, and clinical medicine | New England Journal of Medicine | Obermeyer Z | 2016 | 323 |
| **4** | Big data in health care: using  analytics to identify and manage  high-risk and high-cost patients | Health Affairs | Bates DW | 2014 | 265 |
| **5** | Dermatologist-level classification of skin cancer with deep neural networks | Nature | Esteva A | 2017 | 216 |
| **6** | High-performance medicine: the convergence of human and artificial intelligence | Nature Medicine | Topol EJ | 2019 | 207 |
| **7** | Random forests | Machine Learning | Breiman L | 2001 | 203 |
| **8** | ImageNet classification with deep  convolutional neural networks | Communications of the ACM | Krizhevsky Alex | 2017 | 184 |
| **9** | The parable of google Flu: traps in big data analysis | Science | Lazer D | 2014 | 181 |
| **10** | Big data analytics: Understanding its capabilities and potential benefits for healthcare organizations | Technological forecasting and social change | [Wang, YC](https://webofscience.clarivate.cn/wos/author/record/1690720) | 2018 | 178 |

**Supplementary Figure S1** A visualization network of collaboration between journals.


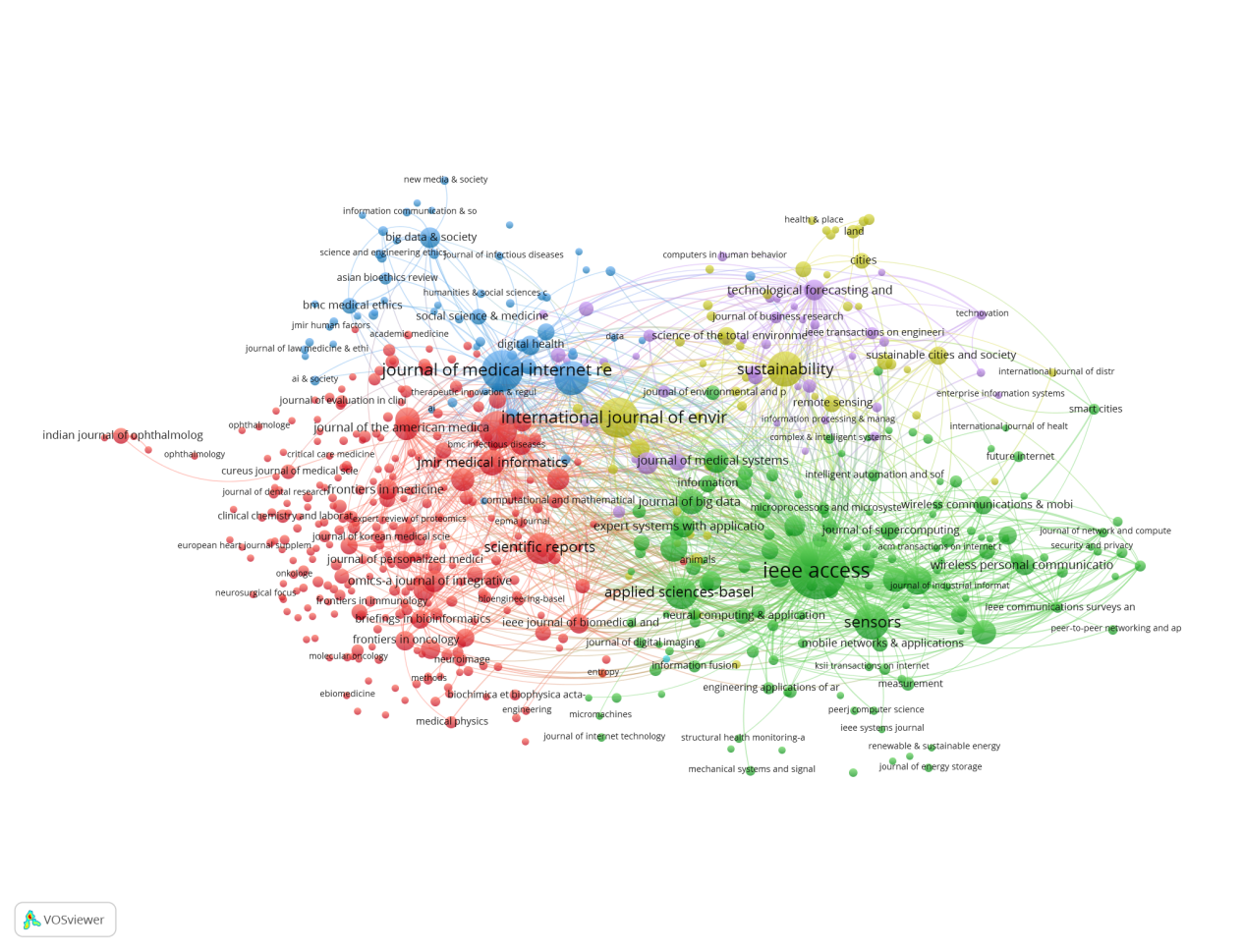


**Supplementary Figure S2** Density map of co-cited journals for big data analytics in health.


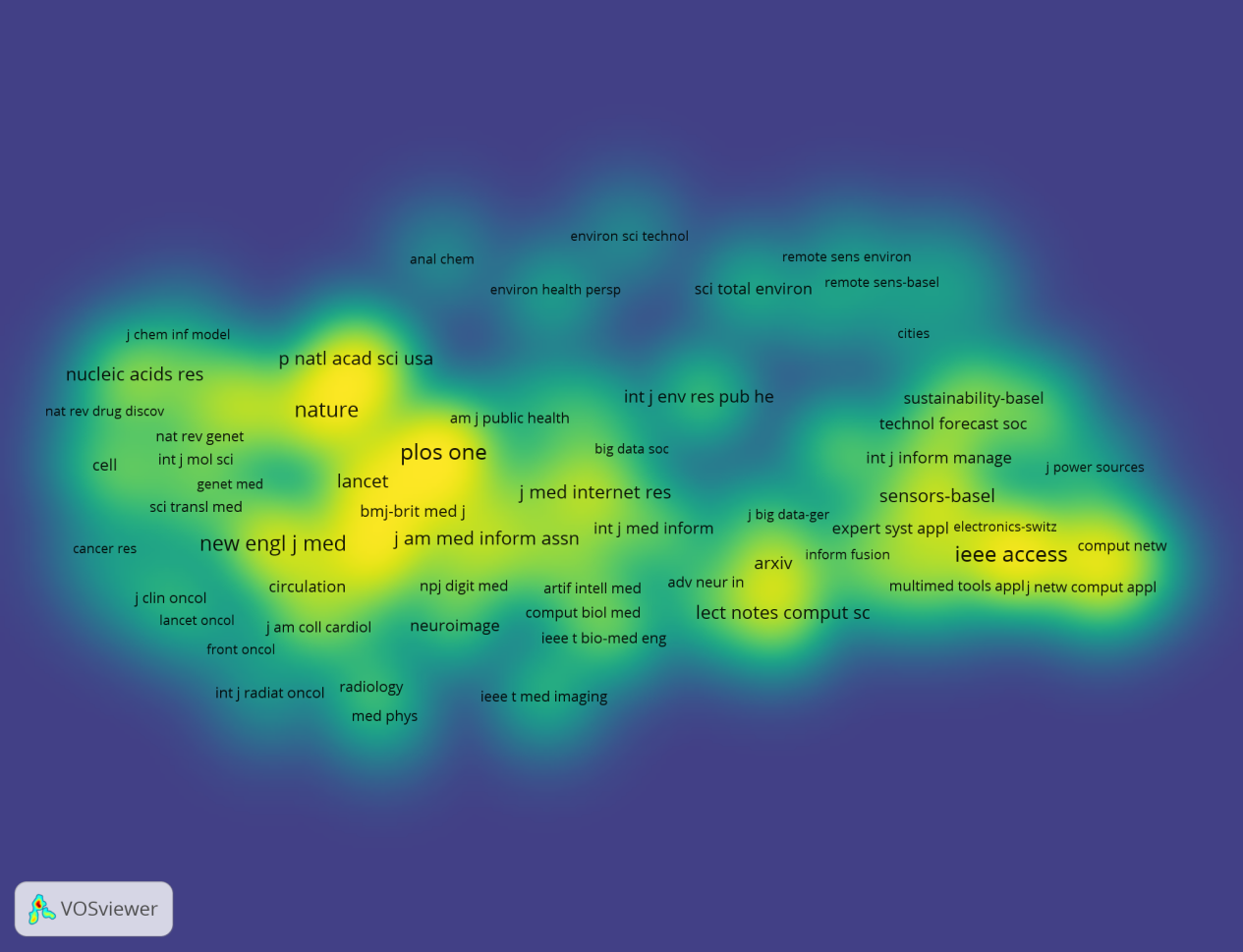


**Supplementary Figure S3** A visualization network of collaboration between authors on big data analytics in health.


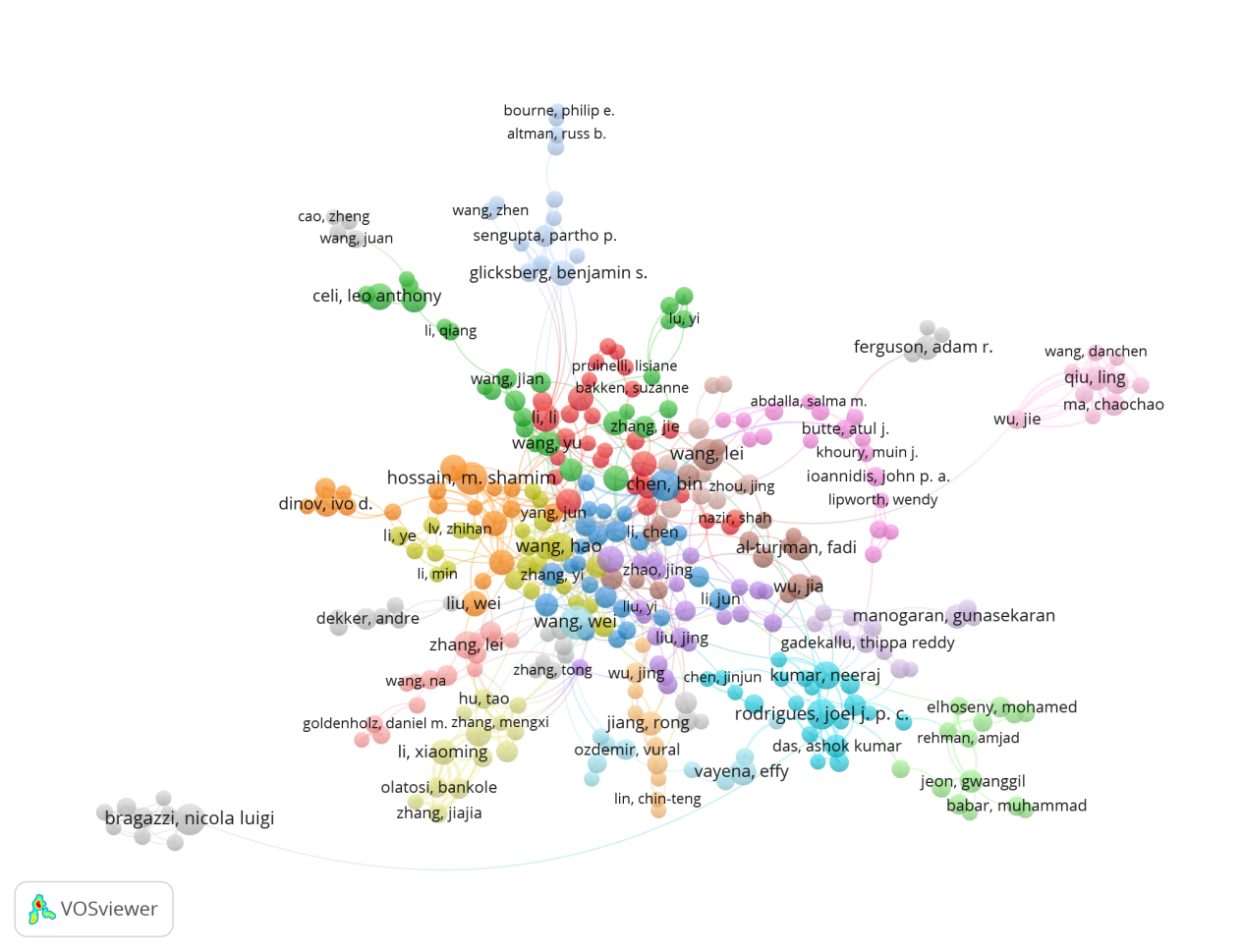


**Supplementary Figure S4** Mapping of co-citation of authors.


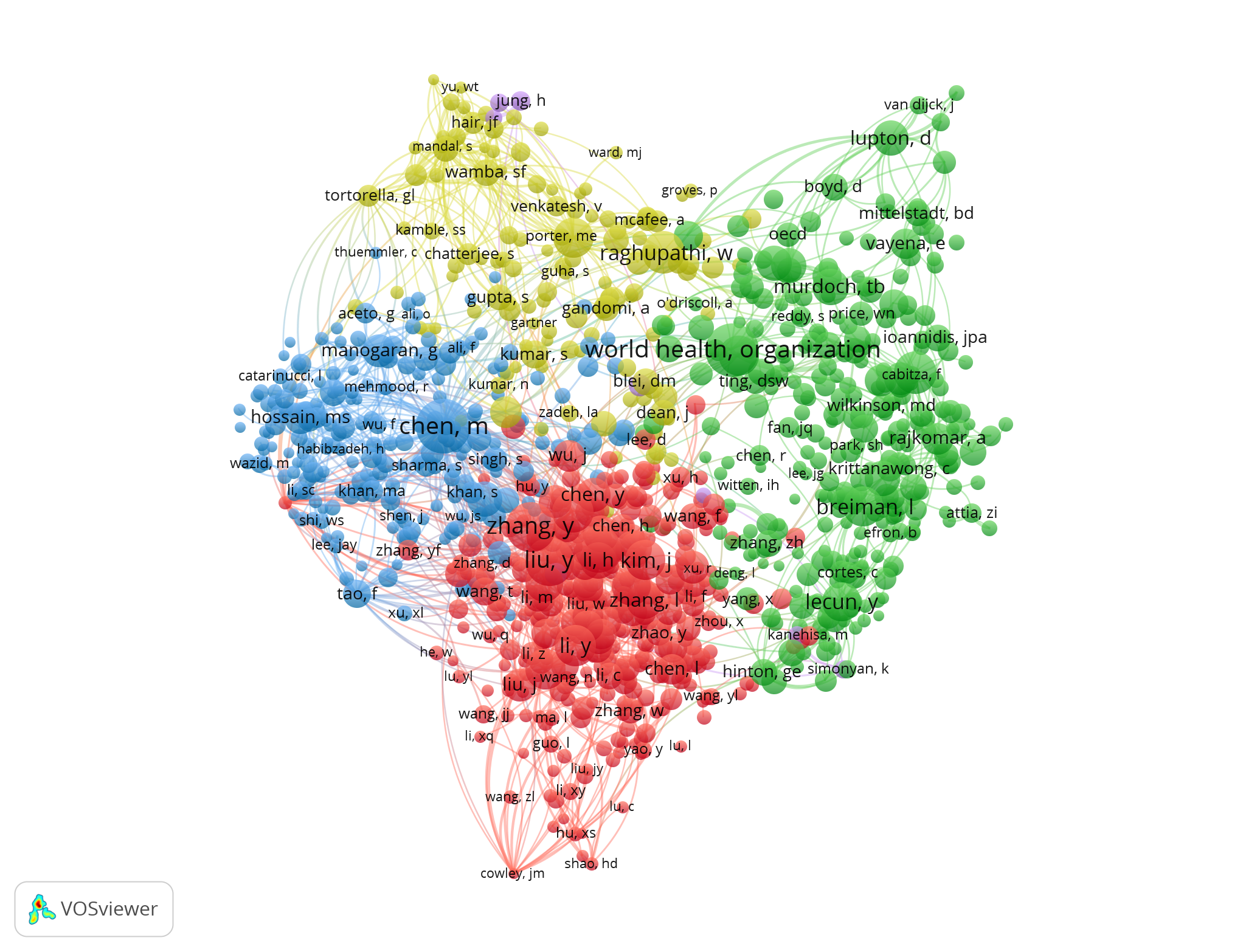


**Supplementary Figure S5** Top 25 reference with the strongest citation bursts.


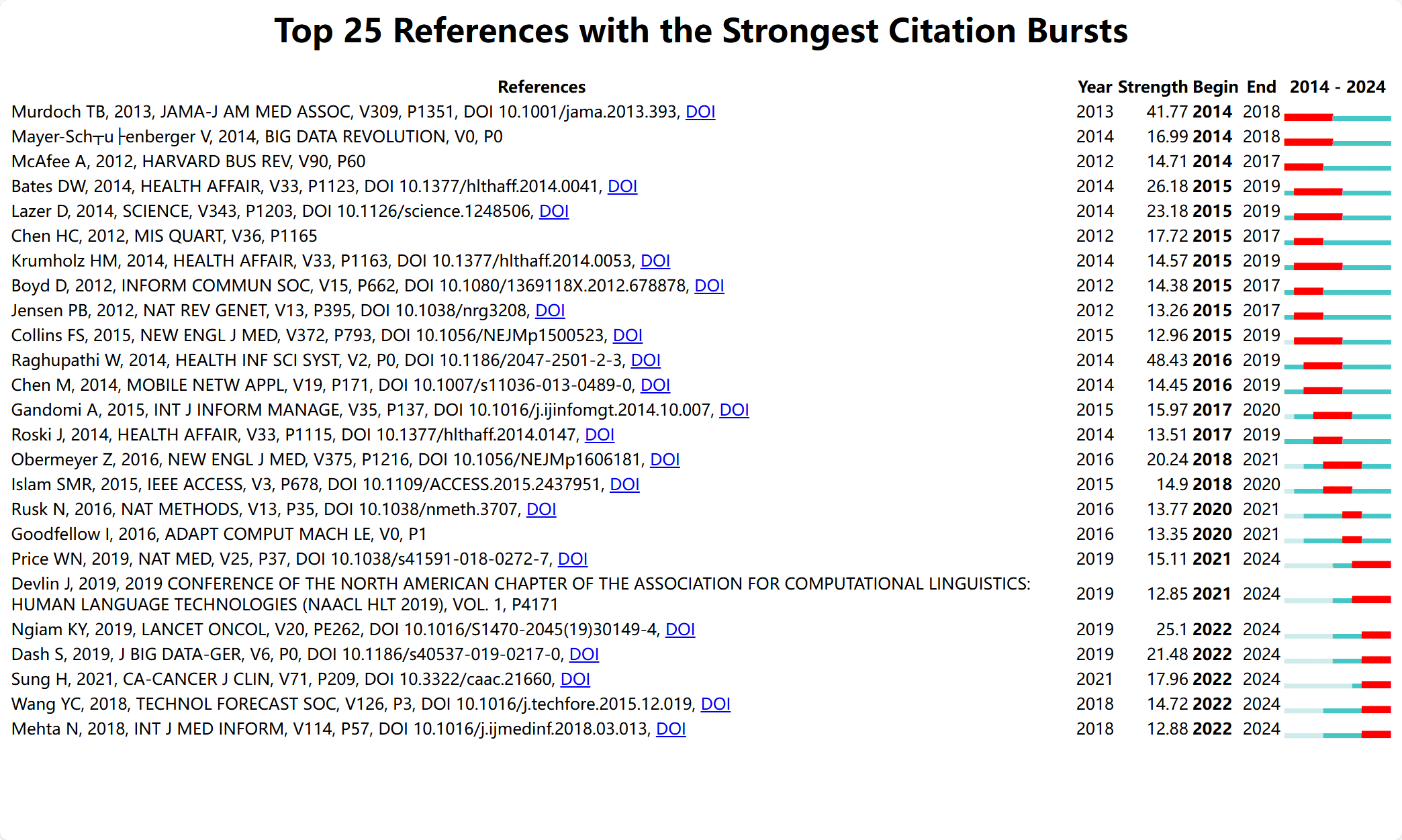


**Supplementary Figure S6** Mapping of keywords overlay.


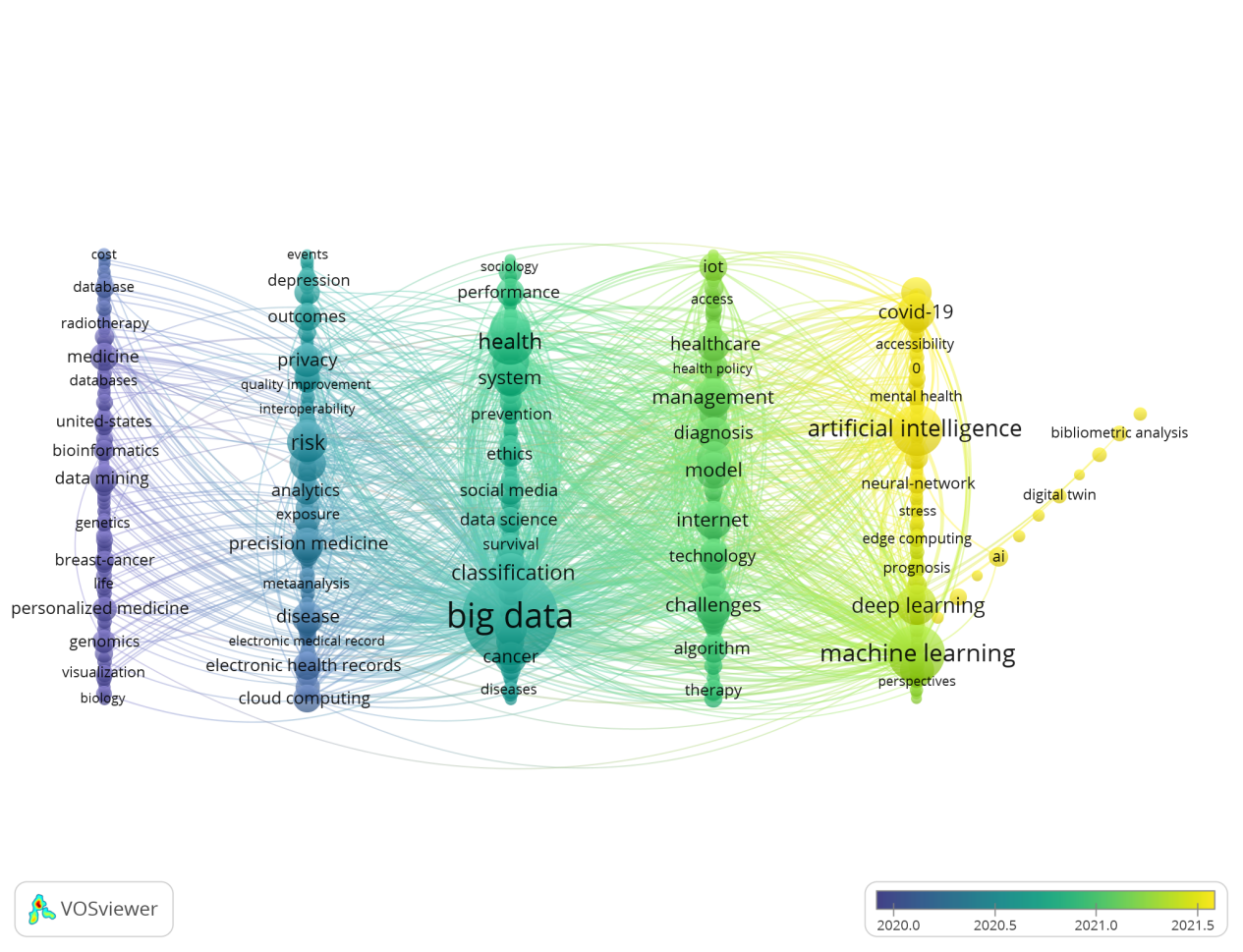

Supplement: Supplementary file 1 [file Data_Sheet_1.docx]
